# A novel technique of using a miniature plate in combination with tension band wiring to treat comminuted patellar fractures

**DOI:** 10.1097/MD.0000000000010311

**Published:** 2018-04-13

**Authors:** Song Gao, Xuqiang Liu, Fengtian Zhang, Tian Gao, Zhihong Zhang, Min Dai

**Affiliations:** Department of Orthopedics, The First Affiliated Hospital of Nanchang University, Artificial Joints Engineering and Technology Research Center of Jiangxi Province, Nanchang, Jiangxi, China.

**Keywords:** comminuted, miniature plate, patella fracture, tension wiring

## Abstract

Traditionally, tension band fixation has been used for treating simple fracture patterns; however, fixation remains a challenge, especially for comminuted fractures. We describe a new method of operation using a combination of a miniature plate with tension band wiring to treat comminuted patellar fractures. The aim of this technique is to transform complicate fractures into simple transverse fractures. As far as we know, no studies using a similar method have been found.

The purpose of this study was to assess the effectiveness of a novel technique in which a miniature plate is used in combination with tension band wiring to treat comminuted patellar fractures.

Between March 2013 and May 2015, 16 patients with closed, displaced, comminuted fractures of the patella were included in the present study. All subjects underwent fixation using a combination of a miniature plate with a tension band wire. Knee function and patient status were evaluated at 12 months using the Böstman knee score and Lysholm knee scale.

The average follow-up period was 15.6 months (range, 12–20 months). At the 12-month follow-up, bone healing was satisfactory in all patients. The average postoperative Lysholm score was 91.6 ± 1.4 (range, 84–97). The average postoperative Böstman scale score was 26.4 ± 0.5 (range, 22–30), thereby indicating excellent results in 4 patients and good results in 12. No patients required reoperation.

The results demonstrate that this new technique is an effective and safe treatment option for comminuted patella fractures, as it is associated with good clinical outcomes.

## 1 Introduction

The patella is one of the thickest articular cartilages in the human body (approximately 5.5 mm), and it plays an important role in the extensor mechanism of the knee. For example, it can increase the moment arm of the extensor mechanism of the quadriceps by 30%.^[1]^ Fracture of the patella is not common, accounting for only approximately 1% of all skeletal injuries.^[2]^ One-third of patella fractures require surgical intervention if the fracture gap exceeds 2 to 3 mm or in patients with joint incongruence.^[3]^ Tension wiring is the most widely used surgical intervention for almost all fracture types. Despite several technical modifications, management of patellar fractures by different types of plate fixation has been reported.^[4–7]^ Operative management of comminuted patella fractures continues to be a challenge with poor functional outcomes and high reoperation rates.

The purpose of the present study was to describe our results with a new technique to preserve the comminuted patella with a combination of using a miniature plate and anterior tension wiring.

## 2 Patients and methods

A total of 16 patients with closed displaced comminuted patella fractures, enrolled between March 2013 and May 2015, were included in the present study. Patients whose patellar articular surface was fractured into more than 3 parts or whose patellar body was fractured into more than 4 parts were considered to have comminuted patellar fractures.^[8]^ Such fractures are defined as displaced fractures with an articular incongruity (step-off) of more than 2 mm or separation of the fragments by more than 3 mm.^[1]^ Patients with concomitant knee fractures, open fractures, or ligamentous or meniscal injuries that required repair were excluded from the study.

This study was approved by the medical ethics committee of The First Affiliated Hospital of Nanchang University concerning the publication of this manuscript and any accompanying images, and all patients provided informed consent.

### 2.1 Surgical technique

All operations were performed under spinal anesthesia. Patients were positioned supine on an operating table, and a midthigh pneumatic tourniquet was used in all patients.

An anterior median incision skin incision (8–10 cm long) was made from the tibial tuberosity to the upper pole of the patella, and subcutaneous dissection was performed. Then, the medial and lateral retinacula were examined, and any tear was enlarged to enable assessment of articular reduction. If no tear was found, lateral arthrotomy was then performed. The fracture hematoma was carefully irrigated, and the fractured fragments were identified. A towel clip was used to fix primary longitudinal fractured fragments, and multiple Kirschner (K)-wires were used to provide temporary fixation. The articular surface congruency was visualized and palpated.

The miniature plate was purchased from Waston Medical (Changzhou, China). A miniature plate with a suitable width and length was placed over the surface of the reassembled patellar surface to transform the complex fractures into simple fractures. The plate was bent slightly if needed, and fixation was achieved using 2.0-mm screws. The articular surface was checked to ensure no penetrating screws. After fixation by screws, some of the K-wires that temporarily fixed the primary longitudinal fractured fragments were removed.

Afterward, we used two 2.0-mm stainless steel K-wires, which ran parallel to each other, to fix the 2 primary fractured fragments in a vertical manner. In some instances, 1.5-mm stainless steel K-wires were used to fix small fractured fragments. The superior and inferior ends of the K-wires protruded by 5 mm, serving as fixation points for the stainless-steel wire, which was laid around the protruding ends of the K-wire, thereby forming a figure-eight configuration on the anterior surface of the patella. The wire was manually twisted until it straightened, and a stable osteosynthesis was achieved.

**Figure 1:**
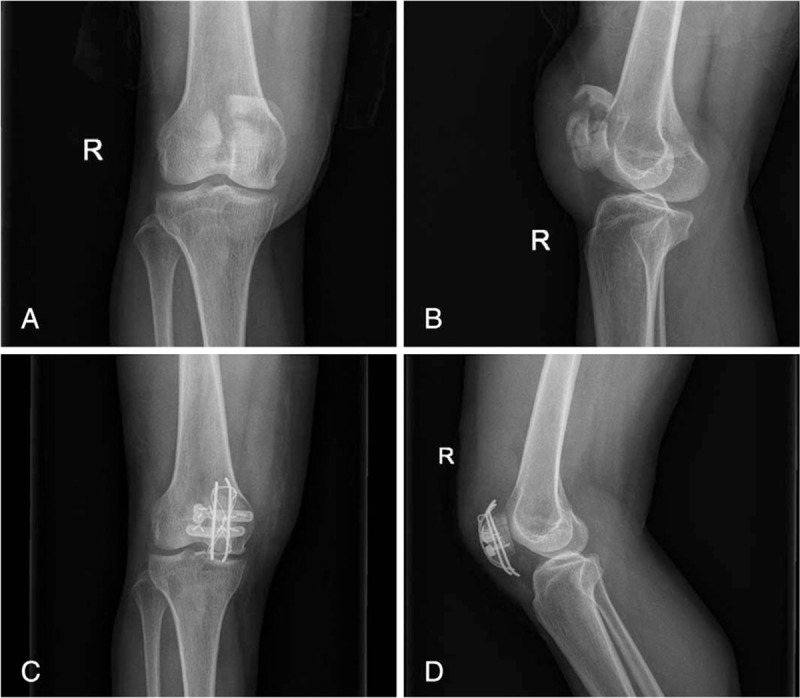
Radiographs of the comminuted patellar fracture (A and B). Radiographs after osteosynthesis (C and D).

Next, the fracture stability was tested intraoperatively through the full range of flexion and extension. The retinacula had been closed using coated Vicryl plus antibacterial sutures (No. 0, Ethicon, Inc., Shanghai, China). Figure 1 A–D indicates preoperative and postoperative radiographs of one of the patients.

### 2.2 Postoperative follow-up

Full weight bearing was allowed as tolerated. The stitches were removed after 14 days. Range of motion exercises were allowed under supervision of a physiotherapist postoperatively to achieve 90° flexion by the end of the third week postoperatively. Radiographs were obtained postoperatively, after 4 weeks, and every 3 months until bone union, and then every 1 year subsequently. At the final follow-up, radiographs were reviewed to record any loosening or hardware failure. A functional knee assessment was conducted using knee range of motion, the Lysholm knee score, and the Böstman knee scale.

## 3 Results

**Figure 2:**
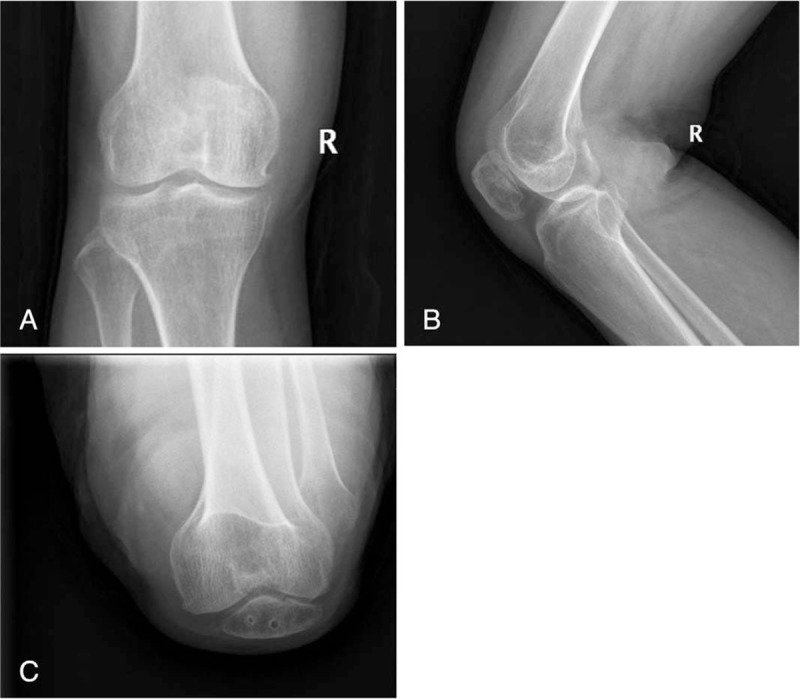
Radiographs after implant removal showing osseous healing of the fragments (A–C).

Twelve women and 4 men were included, with an average age of 48.2 years (range, 29–90 years). The mode of trauma was mainly direct trauma (traffic crash in 7 patients and fall in 9). Full range of knee flexion/extension was achieved in all patients. The average follow-up period was 15.6 months (range, 12–20 months) (Table 1). The costs for plate fixation of patella fracture was 303 USD and the total hospital cost of treatment of a patient suffering from a patella fracture in China was 1.896 USD. At the final follow-up, bone healing was satisfactory in all patients (Fig. 2 A–C). The average postoperative Lysholm score was 91.6 ± 1.4 (range, 84–97). The average postoperative Böstman scale score was 26.4 ± 0.5 (range, 22–30), indicating excellent results in 4 patients and good results in 12. No patients required reoperation (Table 1).

**Table 1:**
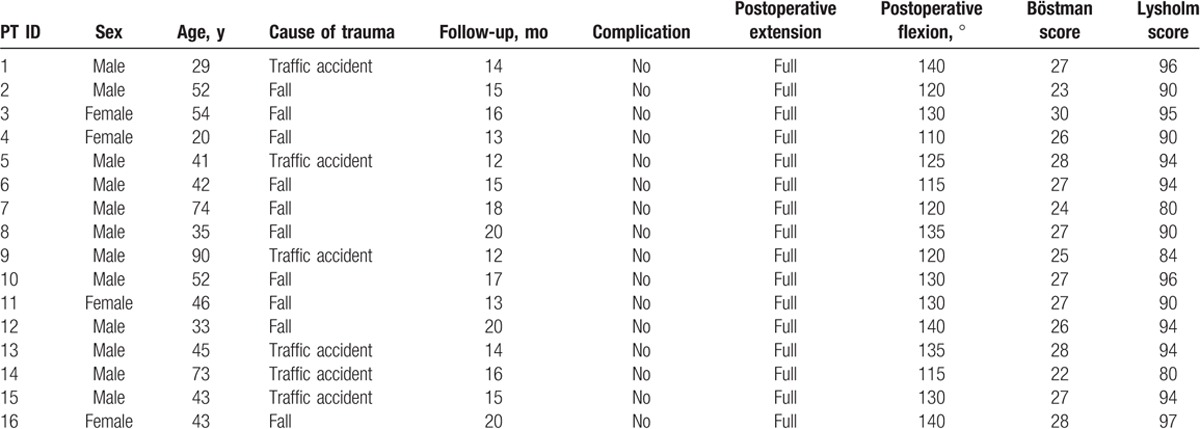
Summary of data from the 16 patients.

## 4 Discussion

Anterior tension wiring using K-wires is still a widely advocated technique for patella fractures.^[9]^ Tension band fixation can successfully treat simple patella fracture patterns. However, when it is used in patients with more complex fractures, especially comminuted fractures, it has been found that the fracture block cannot be sufficiently fixed by the operator during the operation, thereby resulting in extended immobilization and poor functional outcomes.^[10]^ Despite several technical modifications, management of operative patella fractures continues to be a challenge with poor functional outcomes and high reoperation rates.^[1,11–14]^

There are several key advantages to using anterior tension wiring with K-wires for treating comminuted patella fractures include the following. For example, tensile forces can be transformed into compression forces, thereby making it possible for the bone to withstand high tension loads and promote fracture healing. Additionally, it is inexpensive compared with other techniques.^[15]^ The primary disadvantage of this technique is implant-related complications. One study reported a postoperative complication rate of 18%,^[16]^ which included the following: failure of fixation; rupture of a wire used for the tension band due to repetitive motion, resulting in disruption of the fractured site^[17]^; and prolonged recovery and poor function of knee due to the biomechanical limitations of the tension band construct (e.g., early motion is limited to prevent loss of fracture reduction).^[18]^

Despite the high reoperation rate, modified anterior tension wiring is still used. Indeed, it is the most commonly used technique worldwide to treat patella fractures.^[19]^ Therefore, we propose a new method of operation involving an improved tension band fixation technique. We use a combination of a miniature plate with tension band wiring to treat complex patellar fractures. To the best of our knowledge, no studies of a similar method have been published. We first use a miniature plate to fix the longitudinal and oblique fracture (Fig. 1 A–D). This serves to transform complex fractures into simple transverse fractures. Then, tension band wiring is used to fix the transverse fracture.

This new method has many advantages. First, it makes it easy to fix small fractures. Second, it can fix fractured fragments in the coronal plane that anterior tension wiring cannot fix. Third, because the contact surface of the miniature plate is leveled, it can contribute to fracture reduction and articular surface congruency. Fourth, the operation is simple, and the learning curve is short for an experienced surgeon. Fifth, biomechanical studies have shown superior fixation strength with plates compared with tension banding.^[20,21]^ The combination of a miniature plate with tension band wiring can achieve stronger stabilization, and allows the patella to be more evenly stressed and capable of withstanding higher biomechanical loads. This can avoid disruption of the fractured site, migration of a K-wire inserted parallel to the patella, and limitation of early motion postoperatively. Finally, compared to the method of using a combination of lag screws with a tension band, our method can avoid the problem of rotational displacement of the patellar fragment and an uneven joint surface caused when using a lag screw to fix a fragment.

The combination of a miniature plate with tension band wiring preserves the advantages and avoids the disadvantages of tension band wiring alone. This construct allows for stable fixation, osseous union of comminuted patella fractures, reduction of complications, more rigid stabilization and earlier mobilization, decreased knee stiffness, and improved functional outcomes. In our study, no device-related complications were observed.

The main limitation of the present study is the small sample, short-term follow-up and lack of biomechanical analysis. A research study with a bigger sample, longer follow-up and biochemical analysis is in progress, and our findings will be published.

## 5 Conclusion

Using the combination of a miniature plate and anterior tension wiring is an effective, safe treatment option that can be used in the near future for comminuted patella fractures, as it is associated with good clinical outcomes.

## Acknowledgments

We greatly appreciate the assistance and efforts from the staff of the Department of Imaging, The First Affiliated Hospital of Nanchang University.

## Author contributions

**Investigation:** F. Zhang.

**Methodology:** F. Zhang, M. Dai, S. Gao.

**Project administration:** M. Dai, Z. Zhang.

**Resources:** F. Zhang, S. Gao.

**Writing – original draft:** F. Zhang.

**Writing – review & editing:** M. Dai, S. Gao, X. Liu, Z. Zhang.
