# A novel technique of using a miniature plate in combination with tension band wiring to treat comminuted patellar fractures: Erratum

**DOI:** 10.1097/MD.0000000000010684

**Published:** 2018-05-04

In the article, “A novel technique of using a miniature plate in combination with tension band wiring to treat comminuted patellar fractures”,^[1]^ which appears in Volume 97, Issue 15 of *Medicine*, the order of the authors should be “Song Gao, PhD, Fengtian Zhang, BD, Tian Gao, BD, Xuqiang Liu, PhD, Zhihong Zhang, GD, Min Dai, GD.”
